# Cardiovascular Disease and HIV Infection in Sub-Saharan Africa: Misplaced Priorities in the Public Health and Research Agendas?

**DOI:** 10.3389/fcvm.2019.00035

**Published:** 2019-04-05

**Authors:** Luchuo Engelbert Bain, Gerald Chia Gwain

**Affiliations:** ^1^Faculty of Science, Athena Institute for Research on Innovation and Communication in Health and Life Sciences, Vrije Universiteit, Amsterdam, Netherlands; ^2^Department of Behavioral Health, Northwest Hospital, Randallstown, MD, United States

**Keywords:** cardiovascular risk, research, HIV, triple, burden, disease

## Abstract

The effectiveness of combined antiretroviral therapy, not only allows people living with HIV (PLHIV) to live longer, but also exposes them to extra cardiometabolic risks. The recent report of the International AIDS (Acquired Immune Deficiency Syndrome) Society and the Lancet commission are clear on the fact that, the world is not on track regarding ending the HIV/AIDS pandemic by the year 2030 (UNAIDS 90-90-90 targets). We highlight the priority public health interventions, as well as research actions to cope with this almost inevitable triple burden of disease for the continent. Improvement of routine data collection within the health systems strengthening agenda, prioritization of primary health care as the cornerstone of an effective health care system to contain HIV as a chronic disease, and multiplication of large cohort studies in Sub-Saharan Africa are the main public health and research imperatives.

With the advent of combined anti-retroviral therapy, people living with HIV live longer and have their quality of life has improved dramatically. The evidence of increased risk of cardiovascular disease (CVD) among these persons is almost conclusive (1, 2). Triant et al. have recently reported the inaccuracy in HIV infection, of cardiovascular risk prediction functions developed for use in the general population, and highlight the systematic underestimation of cardiovascular risk in a cohort of HIV—infected men (3). Kengne and Ntsekhe (4) point out with the Triant et al. study, the lack of statistical power to reliably validate and update models, failure to include populations with unknown magnitude of HIV related factors to CVD, and suggest that external validation should be ideally conducted by investigators independent from those who developed the model. They highlight the urgency and imperativeness of conducting prospective studies with rigorously collected data to better ascertain CVD risk in its entity in PLHIV. Kengne and Ntsekhe suggest that the risk models should be used across the region until locally relevant evidence becomes available (4). Triant et al included exclusively men in their analysis (3). It is questionable however how the models would be useful and potentially implemented in regions of the world, where most of the care is offered in primary health care facilities, with staff not sufficiently trained to understand, and translate model recommendations into practice.

There are about 37 million persons living with HIV worldwide (5). In 2016, there were 19.4 million people living with HIV (53%) in Eastern and Southern Africa, and 6.1 million (17%) in Western and Central Africa (5). Sub-Saharan Africa represents only 12% of the global population, yet harbors 71% of the global burden of HIV infection (6). cART is important risk factor for developing cardiovascular disease (2, 7–10). Noubiap et al. in a recent systematic review have reported an increased risk and prevalence of dyslipidemia amongst HIV infected persons (2). Arterial damage, especially carotid atheromatosis progression has been shown to be associated with cART exposure (8). Even in short term prospective studies, the increased risk of worsening cardiovascular disease risk profiles is overwhelmingly being showcased (8, 9). Unfortunately, in Sub-Saharan Africa, there are scant data on the risk of cardiovascular disease in HIV-positive populations, and thus, the risk of cardiovascular disease in HIV-infected African patients is largely unknown (7, 11). It is imperative to set up large cohort studies, which include women and children, and persons from diverse backgrounds, especially those from the most infected and affected populations (2, 4, 12), in Africa. It is urgent, and imperative to conduct prospective studies with rigorously collected data to better ascertain CVD risk in its entity in PLHIV (3, 4, 7, 11, 12). There are initiatives underway to fill the HIV prospective study research scarcity in Africa that should be welcome like:

1. The Ndlovu Cohort Study, a prospective study from the Moutse area, Limpopo Province, in South Africa, which shall recruit 1000 HIV-positive and 1000 HIV-negative participants aged 18 years and older (13). The aim of this prospective study is to provide insight into the burden of cardiovascular risk factors and disease, the driving mechanisms, and the contribution of HIV infection and its treatment to the development of CVD in a rural area in Sub-Saharan Africa. The findings from the study are expected to improve upon cardiovascular risk prediction and prevention approaches and models, in HIV infected and non-infected high—risk populations in a resource limited setting.
2. The EndoAfrica study shall provide a unique opportunity to recruit a cohort of HIV-infected patients and HIV-negative controls, who will be comprehensively and longitudinally assessed for cardiovascular risk and disease profile, with vascular endothelial function as a potentially important intermediate cardiovascular phenotype (14). The research group aims to determine how HIV-infection and/or cART are associated with cardiovascular disease and vascular endothelial function, and to compare changes in these parameters at endpoint, 18 months after the initial baseline assessments.

It is idle to think that these prospective studies shall be enough to provide sufficient data, to monitor and evaluate the HIV pandemic in Africa. Initiatives that promote routine quality data collection within the health systems strengthening agenda, especially in Africa, will not only benefit effective research and policy recommendations in PLHIV, but also the entire health needs spectrum. The global health community is not on track as far as ending the HIV pandemic by 2030 (UNAIDS 90-90-90 targets) (15). With HIV becoming a chronic disease, the human resource challenge with scarcity of specialists, mandates African governments to reflect on improving upon primary care. With already stressed health systems from Non Communicable Diseases (Diabetes and Hypertension) due to the demographic transition, the extra burden exerted by HIV on health systems will be a real challenge in the coming years. Empowering primary health care facilities in properly integrating CVD management remains an unavoidable cornerstone in optimally taking care of PLHIV (1). Raising awareness amongst clinical staff to be keener on cardiometabolic disease profiles in PLHIV and to provide timely referrals to specialists in case of need could be a more feasible starting point.

## Author Contributions

LB conceived the initial idea, searched the literature and wrote the initial version of the manuscript. GG provided intellectual input. All authors have read and agreed on the final version of the submitted paper.

### Conflict of Interest Statement

The authors declare that the research was conducted in the absence of any commercial or financial relationships that could be construed as a potential conflict of interest.

## References

[1] Nou E Lo J Hadigan C Grinspoon SK Pathophysiology and management of cardiovascular disease in HIV-infected patients. Lancet Diabetes Endocrinol. (2016) 4:598–610. 10.1016/S2213-8587(15)00388-5 26873066 PMC4921313

[2] Noubiap JJ Bigna JJ Nansseu JR Nyaga UF Balti EV Echouffo-Tcheugui JB. Prevalence of dyslipidaemia among adults in Africa: a systematic review and meta-analysis. Lancet Global Health. (2018) 6:e998–1007. 10.1016/S2214-109X(18)30275-4 30103999

[3] Triant VA Perez J Regan S Massaro JM Meigs JB Grinspoon SK Cardiovascular risk prediction functions underestimate risk in HIV infection. Circulation. (2018) 37:2203–14. 10.1161/CIRCULATIONAHA.117.028975 PMC6157923 29444987

[4] Kengne AP Ntsekhe M. Challenges of cardiovascular disease risk evaluation in people living with HIV infection. Circulation. (2018) 137:2215–7. 10.1161/CIRCULATIONAHA.118.033913 29784678

[5] Global HIV and AIDS Statistics — 2018 Fact Sheet [Internet] Available online at: http://www.unaids.org/en/resources/fact-sheet (accessed November 5, 2018).

[6] Kharsany ABM Karim QA. HIV Infection and AIDS in Sub-Saharan Africa: current status, challenges and opportunities. Open AIDS J. (2016) 10:34–48. 10.2174/1874613601610010034 27347270 PMC4893541

[7] Manga P. HIV and heart disease in Africa. J Am Coll Cardiol. (2015) 66:586–8. 10.1016/j.jacc.2015.06.021 26227198

[8] Psichogiou M Kapelios CJ Konstantonis G Argyris A Nasothimiou E Papadopoulou M. Prevalence, incidence, and contributors of subclinical atheromatosis, arteriosclerosis, and arterial hypertrophy in HIV-infected individuals: a single-center, 3-year prospective study. Angiology. (2019) 70:448–57. 10.1177/0003319718801093 30235944

[9] Cibrián-Ponce A Sánchez-Alemán MA García-Jiménez S Pérez-Martínez E Bernal-Fernández G Castañon-Mayo M. Changes in cardiovascular risk and clinical outcomes in a HIV/AIDS cohort study over a 1-year period at a specialized clinic in Mexico. Ther Clin Risk Manage. (2018) 14:1757–64. 10.2147/TCRM.S170536 30288045 PMC6161730

[10] Hsue PY Waters DD. Time to recognize HIV infection as a major cardiovascular risk factor. Circulation. (2018) 138:1113–5. 10.1161/CIRCULATIONAHA.118.036211 30354392 PMC8063774

[11] Carr A Ory D. Does HIV cause cardiovascular disease? PLoS Med. (2006) 3:e496. 10.1371/journal.pmed.0030496 17132070 PMC1664609

[12] Bain LE Kum AP Ekukwe NC Clovis NC Enowbeyang TE. HIV, cardiovascular disease, and stroke in sub-Saharan Africa. Lancet HIV. (2016) 3:e341–2. 10.1016/S2352-3018(16)30092-3 27470024

[13] Vos A Tempelman H Devillé W Barth R Wensing A Kretzschmar M. HIV and risk of cardiovascular disease in sub-Saharan Africa: rationale and design of the Ndlovu Cohort Study. Eur J Prev Cardiol. (2017) 24:1043–50. 10.1177/2047487317702039 28379043

[14] Strijdom H De Boever P Walzl G Essop MF Nawrot TS Webster I. Cardiovascular risk and endothelial function in people living with HIV/AIDS: design of the multi-site, longitudinal EndoAfrica study in the Western Cape Province of South Africa. BMC Infect Dis. (2017) 17:41. 10.1186/s12879-016-2158-y 28061822 PMC5219697

[15] Bekker L-G Alleyne G Baral S Cepeda J Daskalakis D Dowdy D. Advancing global health and strengthening the HIV response in the era of the Sustainable Development Goals: the International AIDS Society—Lancet Commission. Lancet. (2018) 392:312–58. 10.1016/S0140-6736(18)31070-5 30032975 PMC6323648

